# Comparing strategies for evaluation of candidate genes in case-control studies using family data

**DOI:** 10.1186/1753-6561-1-s1-s31

**Published:** 2007-12-18

**Authors:** Xin Tian, Jungnam Joo, Colin O Wu, Jing-Ping Lin

**Affiliations:** 1 Office of Biostatistics Research, National Heart, Lung and Blood Institute, 6701 Rockledge Drive, Bethesda, Maryland 20892, USA

## Abstract

The goal of this analysis is to compare different test strategies for genetic association in case-control studies using related individuals. The first test is the trend test that is corrected for related individuals on the basis of identity-by-descent information. The second approach is to use generalized estimating equations to adjust for the correlation between relatives, and the third is the multiple outputation method. We compare the power of these test strategies in a simulation study, and apply these methods to a candidate gene dataset of Genetic Analysis Workshop 15 from the North American Rheumatoid Arthritis Consortium.

## Background

The case-control design is a widely used and powerful approach for genetic association studies [1,2]. Genotype frequencies are compared between case and control samples to identify candidate genes or nearby markers that are associated with the susceptibility to a disease. Although association studies may be subject to the possibility of population stratification, it has been recognized that this effect is small in magnitude in well designed studies that sample controls and cases from a homogeneous population, or that match cases by the major confounding variables such as age, gender, and race-ethnicity [1]. Recently, there has been increasing interest in statistical methods that evaluate association between genetic markers and disease status using family-based data [2,3]. This would allow data available from linkage studies or multicase families to be used efficiently to test for association. Unlike traditional case-control studies in which all individuals are unrelated, cases from the same family are often correlated because these individuals share genetic and environmental conditions. Consequently, the frequency of risk alleles at a marker locus is usually increased among related cases relative to unrelated cases. Using related cases sampled from families or ascertained from family linkage studies and unrelated controls may increase the false positive rate (type I error) of an association test, compared to the traditional case-control design based on independent samples. Ignoring the dependence among related individuals may potentially lead to incorrect or spurious results. Hence, any test of genetic association must account for correlation among family members.

Different methods may be used to evaluate genetic associations of candidate genes in case-control studies when some individuals (cases or controls) are related. We briefly sketch three of these methods, the Cochran-Armitage trend test corrected for identity-by-descent (IBD) information, the generalized estimating equations method, and the multiple outputation method. Little is known about their relative efficiency and performance. We compare their power in a simulation study and apply these methods to the candidate gene data of Genetic Analysis Workshop 15 (GAW15) from the North American Rheumatoid Arthritis Consortium (NARAC), which contains affected sibs with rheumatoid arthritis and unrelated controls.

## Methods

### Cochran-Armitage trend test accounting for related individuals

Consider data for a case-control study of genetic association as in Table 1. Assume a marker of a candidate gene with two alleles: *N *and *M*, where *N *is a normal allele and *M *is a risk allele or is in linkage disequilibrium with a risk allele. Denote genotypes as g_0 _= *NN*, g_1 _= *NM*, and g_2 _= *MM*. Let the genotype frequencies for cases and controls be *p*_*j *_and *q*_*j*_, *j *= 0, 1, 2, respectively. Hence, the null hypothesis of no association is *p*_*j *_= *q*_*j *_for each *j*.

**Table 1:** The data in a case-control study

Sample	*NN*	*NM*	*MM*	Total
Case	*r*_0_	*r*_1_	*r*_2_	*R*
Control	*s*_0_	*s*_1_	*s*_2_	*S*
				
Total	*n*_0_	*n*_1_	*n*_2_	*n*

Given the data in Table 1, the Cochran-Armitage trend test for association [4] between a disease and a marker can be written as *Z*_*x *_= *U*(*x*)/$\hat{\sigma }$, where *U*(*x*) = $n^{-1}\sum_{j=0}^{2}x_{j}(Sr_{j}-Rs_{j})$, and *x *= (*x*_0_, *x*_1_, *x*_2_)^*T *^is a set of increasing scores (weights) assigned to the three genotypes (g_0_, g_1_, g_2_) *a priori *based on the underlying genetic model. Under the null hypothesis, $\mathrm{var}[U(x)]=n^{-1}RS[\sum_{j=0}^{2}x_{j}^{2}p_{j}-{\left(\sum_{j=0}^{2}x_{j}p_{j}\right)}^{2}]$, which can be estimated by ${\hat{\sigma }}^{2}=n^{-3}RS[n\sum_{j=0}^{2}x_{j}^{2}n_{j}-{\left(\sum_{j=0}^{2}x_{j}n_{j}\right)}^{2}]$; *Z*_*x *_asymptotically follows a standard normal distribution *N*(0, 1).

However, because cases and controls within the same family may be biologically related, Slager and Schaid [3] proposed the following method for estimating the variance to account for correlations among related cases or controls. Let *u*_*i *_= (*u*_*i*0_, *u*_*i*1_, *u*_*i*2_)^*T *^be the genotype indicator vector for the *i*^th ^case, where *u*_*ij *_= 1 for the *i*^th ^case with genotype g_j _and *u*_*ij *_= 0 otherwise, *i *= 1,...,*R*. Similarly, we use *v*_*j *_for controls. Then $r={\left(r_{0},r_{1},r_{2}\right)}^{T}=\sum u_{i}$, and $s={\left(s_{0},s_{1},s_{2}\right)}^{T}=\sum v_{j}$. Let *φ *= *R*/*n*. Then the above test statistic is *U*(*x*) = *x*^*T *^[(1 - *φ*)*r *- *φs*], and var[*U*(*x*)] = *x*^*T*^{var[(1 - *φ*)*r *- *φs*]}*x *= $x^{T}\{{\left(1-\varphi \right)}^{2}\mathrm{var}(\sum u_{i})+\varphi^{2}\mathrm{var}(\sum v_{j})-2\varphi (1-\varphi )\mathrm{cov}(\sum u_{i},\sum v_{j})\}x$.

Here the variance and covariance terms can be calculated based on the multinomial distributions and IBD-sharing probabilities for pairs of related individuals [3].

### Generalized estimating equations (GEE) method

The GEE developed by Liang and Zeger [5] for the analysis of longitudinal data can be applied for case-control data in genetic studies. Let $y_{i}={\left(y_{i1},...,y_{i,n_{i}}\right)}^{T}$ be the response variable for *n*_*i *_related subjects, *i *= 1,...,*m*, where *m *is the total number of families. For a binary trait, *y*_*ij *_= 1 for cases and 0 for controls. The logistic regression model can be considered for the case-control data in Table 1: log[*E*(*y*_*ij*_)/(1 - *E*(*y*_*ij*_))] = *β*_0 _+ *β*_1_*x*_*ij *_+ $\beta_{2}^{T}$*w*_*ij*_, where *x*_*ij *_= *x*_0_, *x*_1_, or *x*_2 _is the score assigned to the genotype as above, and *w*_*ij *_denotes other covariates. The test of genetic association is equivalent to the test of *β*_1 _= 0. Due to correlation of related family members, the conventional methods assuming independence are incorrect. The estimate and standard error for *β *= (*β*_0_, *β*_1_, $\beta_{2}^{T}$)^*T *^based on the GEE procedure take into account the within-family correlation, where *β *is estimated by solving the equations $\sum_{i=1}^{m}{\left(\frac{\partial \mu_{i}}{\partial \beta }\right)}^{T}V_{i}^{-1}(y_{i}-\mu_{i})=0$, with *μ*_*i *_= *E*(*y*_*i*_; *β*) and *V*_*i *_= *V*_*i*_(*y*_*i*_; *β*, *θ*) denoting the "working" covariance matrix of *y*_*i*_. The estimate of *β *is asymptotically normally distributed and its variance is given by $\Sigma =\Sigma_{1}^{-1}\Sigma_{2}\Sigma_{1}^{-1}$, where $\Sigma_{1}=\sum_{i=1}^{m}{\left(\frac{\partial \mu_{i}}{\partial \beta }\right)}^{T}V_{i}^{-1}(\frac{\partial \mu_{i}}{\partial \beta })$, and $\Sigma_{2}=\sum_{i=1}^{m}{\left(\frac{\partial \mu_{i}}{\partial \beta }\right)}^{T}V_{i}^{-1}(y_{i}-\mu_{i}){\left(y_{i}-\mu_{i}\right)}^{T}V_{i}^{-1}(\frac{\partial \mu_{i}}{\partial \beta })$. There are a number of choices for *V*_i _and it has been shown that the GEE estimates are valid and consistent even if the working covariance matrix is misspecified. For family or affected sib-pair data, a simple and reasonable choice is the exchangeable correlation matrix with a common correlation *θ *for each pair of relatives [6].

### Multiple outputation (MO) method

The MO method proposed by Hoffman et al. [7] and Follmann et al. [8] provides inferences for clustered correlated data by averaging analyses of independent data. For independent case-control data in genetic studies, several methods can provide a normally distributed statistic, $\hat{\beta }$, for the genetic association and an estimate of its variance, $\hat{\sigma }$^2^. For example, the trend test statistic *Z*_*x *_above is a sensible choice, which estimates the weighted differences of the genetic frequencies. For case-control data sampled from families, a new sample can be obtained by randomly selecting an individual from each family, and then $\hat{\beta }$ and $\hat{\sigma }$^2 ^can be computed based on this new sample. After repeating this multiple times, the estimate of association will be the average of the $\hat{\beta }$ values, and an estimate of its variance is given by the average of the $\hat{\sigma }$^2 ^minus the sample variance of the $\hat{\beta }$ values. The MO estimate has been shown to be asymptotically normally distributed.

## Results

### A simulation study

To compare the performance of the three methods, we conducted a small simulation by generating case-control data sets and computing the empirical power for all the tests under three genetic models: recessive, additive, and dominant. The simulations were similar to those performed by Tian et al. [9] with 10,000 replications. We assume that the disease prevalence, *K*, is 0.1, the marker allele frequency, *p*, is 0.3, and Hardy-Weinberg equilibrium holds. To facilitate the calculation, each case-control data set included 200 cases generated as 100 affected sib pairs drawn from 100 different families, and 200 unrelated controls. Let the genotype relative risks *RR*_1 _= f_1_/f_0_, and *RR*_2 _= f_2_/f_0_, where f_0_, f_1 _and f_2 _are the penetrances for genotypes g_0_, g_1_, and g_2_. Thus, equivalently, the null hypothesis can be written as *RR*_1 _= *RR*_2 _= 1. The alternative hypothesis can be specified by varying *RR*_1 _and *RR*_2_.

Table 2 displays the empirical power of the trend test with variance corrected by IBD information (*Z*_IBD-Tr_), the tests based on the GEE estimate (*Z*_GEE_), and the MO estimate (*Z*_MO_). The relative risks *RR*_1 _and *RR*_2 _were chosen so that a particular trend test had about 85% power for each given model. The scores (*x*_1_, *x*_2_, *x*_3_) = (0, 1, 2) for the additive model were used for the three tests in the simulations assuming the underlying model was unknown. Under the null hypothesis of no association, all three tests have the correct type I error, around 0.05. For all three genetic models, both the GEE and MO tests have relatively good power, ranging from 73% to 84%, compared with the IBD-corrected trend test.

**Table 2:** The empirical power of the three tests

Model	(*RR*_1_, *RR*_2_)	*Z*_IBD-Tr_	*Z*_GEE_	*Z*_MO_
Null	(1, 1)	0.05	0.05	0.05
Recessive	(1, 2.54)	0.69	0.73	0.73
Additive	(1.66, 2.32)	0.85	0.82	0.84
Dominant	(1.92, 1.92)	0.80	0.81	0.79

### Application

The GAW15 NARAC candidate gene data consisted of affected sibs with rheumatoid arthritis from multiplex families and unrelated controls. The candidate gene data from the *PTPN22 *locus [10] had 14 SNPs genotyped on 1269 cases and 1519 unrelated controls. The cases were from 665 families: 123 families had 1 case, 492 families had 2 affected siblings, and 50 families had 3 or more affected siblings. For sib pairs from the same family, their IBD sharing probabilities were calculated using the software MERLIN [11].

Table 3 presents results based on the three testing methods and the trend test without adjusting for correlated cases. The performance of these tests is comparable. The Bonferroni correction was applied to adjust for multiple testing of 14 SNPs, and only the SNPs with an adjusted *p*-value less than 0.05 in any one of the tests are presented. All three test methods identified the same markers that were significantly associated with the susceptibility of rheumatoid arthritis. The unadjusted trend test that assumed independent cases overestimated the association and could result in a larger false-positive rate.

**Table 3:** Results for the NARAC candidate gene data

Markers	*Z*_IBD-Tr _(*p*)	*Z*_GEE _(*p*)	*Z*_MO _(*p*)	*Z*_Unadjusted _(*p*)
rs2476601	-7.62 (<10^-6^)	-7.54 (<10^-6^)	-7.81 (<10^-6^)	-8.61(<10^-6^)
rs1217413	4.36 (1.3 × 10^-5^)	4.41 (1.0 × 10^-5^)	4.37 (1.2 × 10^-5^)	4.94 (<10^-6^)
rs1310182	-4.19 (2.8 × 10^-5^)	-4.25 (2.1 × 10^-5^)	-4.23(2.3 × 10^-5^)	-4.76 (1.9 × 10^-6^)
rs1217388	3.40 (0.0007)	3.42 (0.0006)	3.42 (0.0006)	3.84 (0.0001)
rs2488458	3.33 (0.0009)	3.36 (0.0008)	3.41 (0.0007)	3.76 (0.0002)

## Discussion

We consider three methods that use completely different approaches to account for correlation among family members. The IBD-corrected trend test requires the genotype information from parents or other family members to obtain more accurate IBD calculation. Because the variance of the test is corrected for correlation among related cases using the genealogy and marker information, this test is expected to be more powerful than the tests using only family pedigree information. The GEE approach estimates the correlation among related cases through a working correlation matrix, and the MO accounts for the correlation through repeated sampling. In our simulation study, the GEE and MO approaches appear to have similar power. Note that Follmann et al. [8] showed that the GEE estimates under an exchangeable working correlation performed better than MO in some simulations; however, the GEE may have problems converging. They also showed that in certain simple settings MO was slightly more powerful than or competitive to GEE with working independence correlation. The relative efficiency of these tests was unknown in general, and it would require a more extensive simulation to explore their behaviors. In addition, compared to the IBD-corrected trend test, both GEE and MO are simple and broadly applicable approaches that can also easily adjust for multiple covariates.

Note that these methods used in case-control studies are sensitive to population stratification. In genetic association studies, case-control and family-based designs are two fundamentally different approaches. While case-control designs study the contrast of allele/genotype frequencies between cases and controls to identify associations within populations, family-based designs use families to look for susceptibility alleles through transmission within families. Thus, when population stratification is suspected, family-based designs are preferred to case-control designs. For such designs, the well known transmission disequilibrium test (TDT) and its various extensions, such as the family-based association tests (FBATs), are commonly used [2,12]. They are robust against population substructure. However, trios consisting of an affected child and parents are needed for TDT, which may be difficult to obtain. Other designs such as affected sibs and discordant sib pairs have been shown to be less powerful than case-control studies for both rare and common diseases [2,3]. Moreover, to test bi-allelic markers like SNPs, family-based tests require a large number of families because they discard all the homozygous (non-informative) parents. For the above GAW15 example, most of parental genotypes and unaffected siblings are not available for the NARAC candidate gene data. Thus, this data set is not suitable for using either the TDT or FBAT tests. Therefore, when there is no evidence of major population substructure, the cases collected from families for linkage studies can be recycled for association, and additional unrelated controls may be obtained and genotyped to increase the power to confirm the candidate marker.

The test results from the three methods depend on the scores assigned to the genotypes based on the assumption of the underlying genetic models such as recessive, additive, and dominant. In practice, since the genetic model is unknown for most complex diseases, the additive model is usually assumed first, with *x *= (0, 1, 2) indicating the numbers of risk alleles. Applying a trend test with one set of scores would result in a loss of power if the genetic model is misspecified. Hence, more robust tests can be considered to protect against model uncertainty [9].

## Conclusion

In summary, we compare three methods of testing genetic association for case-control studies with cases drawn from families and unrelated controls. Our results indicate that all three methods perform well, and their performance is comparable in the simulation and application to the GAW15 NARAC data. All three methods can be applied to more general situations where the controls or both cases and controls are also correlated.

## List of Abbreviations

FBAT: Family-based association test

GAW: Genetic Analysis Workshop

GEE: Generalized estimating equation

IBD: Identical by descent

MO: Multiple outputation

NARAC: North American Rheumatoid Arthritis Consortium

SNP: Single-nucleotide polymorphism

TDT: Transmission-disequilibrium test

## Competing interests

The author(s) declare that they have no competing interests.
